# Truss for the Permanent Cure of Hernia

**Published:** 1834

**Authors:** 


### VII.—Stagner's Patent Truss for the permanent Cure of Hernia.

In our last number we gave a brief notice of this instrument, the invention of a citizen of Madison county, Kentucky. We have heard that the following circumstance gave rise to the invention. Mr. Stagner having himself a rupture, and losing, one day, a considerable part of the pad of his truss, he resorted to a chip or piece of wood, as a temporary substitute. He soon found, however, that this *hard* body served a better purpose in preventing the descent of the hernia, than the soft pad, and was induced to continue it. After having worn it for a time, he was surprised to find, that when it was off, the rupture did not show it itself; and, at length, had the gratification to discover, that a permanent cure had been effected.

Encouraged by this unexpected result, he turned his mind upon the construction of wooded truss heads, as substitutes for those of softer materials; and has satisfied himself that the form of half an egg, slightly compressed, the division being

10*

made longitudinally, is the most proper. The plain or flat surface is screwed to the spring at the usual place of the pad, and the truss is then applied in the ordinary way, with the parabolic projection directed upon the aperture. The pressure which is thus made, seems to excite about the abdominal ring, a mild, adhesive inflammation, which in a few weeks terminates in the obliteration of the canal.

Several respectable physicians and intelligent citizens of Kentucky, have certified, that within their observation, a number of cases of hernia have been already permanently cured; and we are assured, that in many parts of that state, the invention is considered as entirely adequate to the object proposed.

Mr. Wm. Shadburne, a student of medicine, in the office of Dr. Wood, corner of Fourth and Walnut streets, in this city, is the agent of the patentee, for the city and county. In no instance is a charge made, unless a permanent cure be effected. Should any portion of the omentum adhere from previous inflammation, below where the truss must be applied, a cure cannot of course be effected.

